# Identity Matching and Stimulus Equivalence Learning Paradigms for Memory Rehabilitation of Explicit Memory Deficits: A Scoping Review

**DOI:** 10.1007/s40614-025-00475-z

**Published:** 2025-10-06

**Authors:** Josefine Schedlowski, Joseph H. R. Maes, Ruth J. van Asselt, Dirk Bertens, Jos I. M. Egger, Roy P. C. Kessels

**Affiliations:** 1 Donders Institute for Brain, Cognition, and Behaviour, Radboud University, Nijmegen, The Netherlands; 2 Centre of Excellence for Korsakoff and Alcohol-related Cognitive Disorders, Vincent van Gogh Institute for Psychiatry, Venray, The Netherlands; 3 Klimmendaal Rehabilitation Center, Arnhem, The Netherlands; 4 Radboudumc Alzheimer Centre, Radboud University Medical Centre, Nijmegen, The Netherlands; 5 Centre of Excellence for Neuropsychiatry, Vincent Van Gogh Institute for Psychiatry, Venray, The Netherlands; 6 Tactus Addiction Care, Deventer, The Netherlands

**Keywords:** Stimulus equivalence learning, Explicit memory deficits, Alzheimer’s dementia, Memory rehabilitation, Differential outcome procedure, Relational learning

## Abstract

Explicit memory dysfunction, such as in Alzheimer’s dementia, impairs learning and daily functioning, requiring effective rehabilitation strategies to promote functional independence. Relational learning paradigms such as stimulus equivalence learning (SEL) imply the formation of networks of relations in which trained relations give rise to emergent relations, potentially providing a novel approach to addressing deficits in remembering and stimulus control. We evaluated the scope and nature of research on the application of relational learning paradigms for memory rehabilitation. In particular, we outline the evidence for the efficacy of identity matching and SEL in specific disorders, the associated effective strategies, and challenges to guide future research. A systematic search following the PRISMA-ScR guidelines identified 23 reports categorized into identity matching, arbitrary matching, and differential outcome procedure (DOP) paradigms. Findings were mixed regarding the success of training procedures. Studies indicate particularly positive outcomes under the DOP and overall efficacy seemed to depend on impairment severity. However, current evidence on the efficacy of relational learning paradigms in individuals with explicit memory dysfunction remains inconclusive due to uncontrolled designs and methodological weaknesses in statistical analysis and patient reporting. Nevertheless, insights from the reviewed studies can inform more rigorous future research. The focus should be on identifying the necessary and sufficient conditions for training stimulus equivalence relations in this population, within meaningful and well-controlled experimental designs to validate the preliminary findings and assess SEL’s potential as a cognitive intervention.

The rise in global life expectancy has led to an aging population in the Western world. This demographic shift is accompanied by an increased prevalence of age-related health conditions, such as cognitive impairments due to neurodegenerative disease that may eventually result in dementia. At present, over 57.4 million people worldwide are living with dementia, a number expected to triple by 2050 (Nichols et al., 2022), with the majority having Alzheimer’s dementia (Qiu et al., 2009; Scheltens et al., 2016). One of the most profound features of Alzheimer’s dementia is severe anterograde memory dysfunction, that is, impairments in the ability to learn new information (Markowitsch & Staniloiu, 2012; O’Connor & Race, 2024; Weintraub et al., 2012). Similar explicit memory dysfunction is also observed in conditions such as Korsakoff syndrome, acquired brain injury, and mild cognitive impairment (Kopelman, 2002).

From a behavioral perspective, explicit memory dysfunctions describe a failure to respond accurately under conditions that previously evoked correct responses. In other words, the individual is unable to respond correctly in the presence of a discriminative stimulus. That is, past learning fails to establish or maintain stimulus control over behavior. The consequences of explicit memory dysfunction in daily living are profound, affecting both the individual and their family (Ballard, Boyle et al., 1996a, Ballard, Bannister et al., 1996b; Crellin et al., 2014; Murray et al., 1999). Patients show poor discriminatory behavior, and as a consequence, limitations in functional skills. Successfully engaging in daily routines, expressing the correct names for familiar faces, and failure to take medicine at the appropriate time are examples of deficits that eventually contribute to reduced functional independence and increased social isolation. Given the significant impact of memory dysfunction, it is essential to promote reablement and to develop effective rehabilitation strategies (Poulos et al., 2017). These may improve the quality of life for those affected, their families, and communities, especially in the early stages of the underlying neurodegenerative disease, when symptoms are still mild.

Rehabilitation for remediating deficits in stimulus control and remembering includes a variety of approaches, ranging from holistic interbehavioral interventions to techniques that target stimulus–stimulus and stimulus–response relations. Approaches typically address multiple aspects such as tacting, orienting behavior, social interaction, self-care routines, physical activity, and environmental modifications. The shared goal is the maintenance of current levels of functioning, reducing problem behavior, and increasing reinforcement (Buchanan et al., 2011; Dröes et al., 2011). From a social perspective, therapies such as reminiscence and music therapy often use photographs or familiar songs to evoke past experiences. Environmental modifications aim to enhance discriminative control by arranging stimuli in ways that encourage appropriate responding. Structured environments, visual prompts, external cues (e.g., calendars and notes), and routine cues can thereby increase independent functioning (Buchanan et al., 2011; Lin et al., 2020). Although such aids can be effective in individuals with relatively intact stimulus control, more advanced impairments often require increased external support from caregivers to maintain correct responding.

Cognitive rehabilitation strategies involve behavioral techniques such as spaced retrieval, vanishing cues, and errorless learning (De Vreese et al., 2001; de Werd et al., 2013; Kessels & de Haan, 2003; Ptak et al., 2010). These methods aim to establish stimulus control over specific associations (e.g., object–name pairs) and routines through shaping, chaining, and the avoidance of errors during acquisition. Although these strategies have demonstrated efficacy, their effects are often material- and context-specific, require extensive effort, and may not be maintained over time (Schnider & Ptak, 2023). The focus lies on acquiring single pieces of information, overlooking the integration of broader knowledge networks and more complex tasks. In particular, a person may learn a fact yet may fail to apply it in daily living. Patients may successfully acquire the name of an object (e.g., “pen”), but fail to associate it with its function (e.g., writing) or its context, reducing its practical usefulness. New information needs to be linked to meaningful context and previous knowledge (Schnider & Ptak, 2023). Therefore, methods are needed to establish broader networks of relations between stimuli and events (e.g., linking “faces,” “names,” and “relationships”) to reduce effort and promote generalization.

One such method to establish such relations between stimuli is stimulus equivalence learning (SEL)—a technique rooted in behavioral analysis and first described by Sidman (1971). At its core, SEL works through conditional discrimination training. This is typically implemented within a matching-to-sample task (MTS), through which stimuli gradually become members of an equivalence class, meaning they are functionally grouped despite potential differences in category or modality. An example of trained and derived relational responding is depicted in Fig. 1. Consider three types of stimuli: the written word “Anna” (A), a picture of a person (B), and the written word “daughter” (C). Using an MTS task, initial training establishes conditional relations between pairs (e.g., A–B and A–C), after which untrained relations are tested, which are relations that arise in the absence of reinforcement (e.g., B–C). This testing of untrained relations examines reflexivity (A = A, B = B, C = C), symmetry (e.g., B = A, C = A), and transitivity (e.g., B = C, C = B). Successful performance across these tests confirms the formation of a stimulus equivalence class (Sidman, 2000; Sidman & Tailby, 1982).

**Fig. 1:**
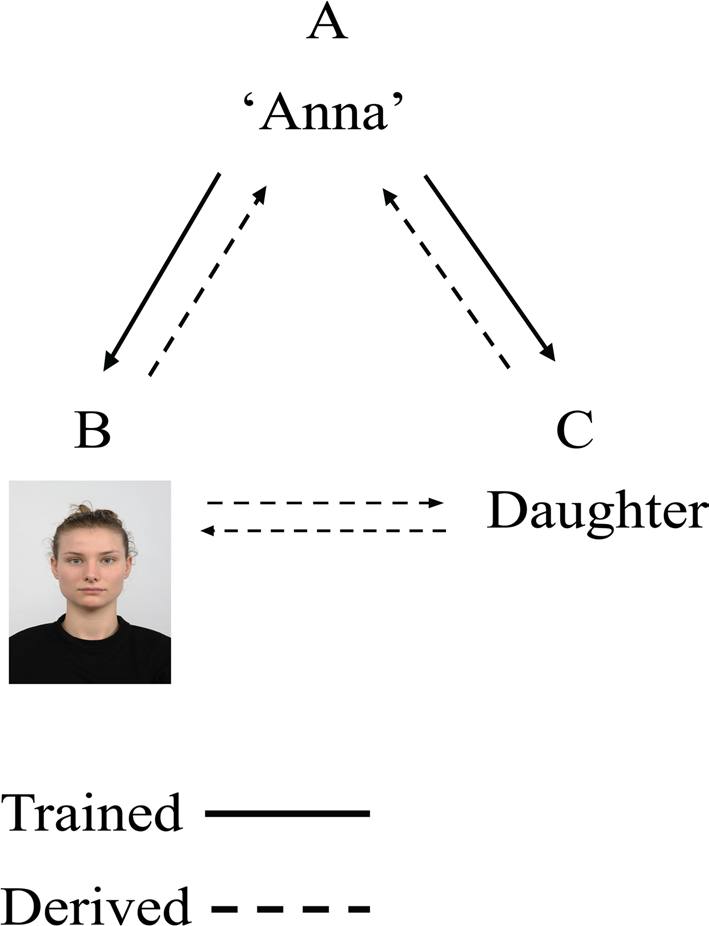
Establishing Equivalence Classes: Trained and Derived Relations. *Note.* Training conditional relations between pairs (e.g., the name of the person in the picture is Anna (A–B) and Anna is a daughter [A–C]) can lead to the emergence of derived relations, such as symmetry (B = A, C = A) and transitivity (the person in the picture is a daughter (B = C and C = B). Successful performance on tests assessing both trained and derived relations indicates the formation of equivalence classes

Adopting the SEL paradigm involves forming networks of trained relations, which may give rise to functionally useful, untrained relations. This reduces the need for direct reinforcement of each relation, making learning more efficient and generalizable across contexts. A simpler variant within the broader category of relational learning is identity matching, which uses stimuli with identical or highly similar features as samples and comparisons. Although identity matching typically does not lead to emergent relations, it can assess discrimination abilities, recognition, and basic stimulus control. It is often viewed as a prerequisite for stimulus equivalence learning (e.g., testing reflexivity) and is especially valuable in the context of more severe behavioral impairments.

There is an abundance of methodological variations and applications of relational learning paradigms across diverse age groups and clinical populations (Aggio et al., 2021; Arntzen, 2012; Carr et al., 2000; Cowley et al., 1992; McLay et al., 2013; Regaço et al., 2023; Shawler et al., 2023). For example, studies within this paradigm have consistently demonstrated success in establishing stimulus equivalence classes in neurodevelopmental disorders such as autism and have also gained attention in school settings through the paradigm’s use in evidence-based instruction, highlighting its broad applicability (McLay et al., 2013; Shawler et al., 2023). Its robustness has been demonstrated across various modalities, including visual, auditory, and olfactory stimuli (Annett & Leslie, 1995; Arntzen & Eilertsen, 2020; Hill et al., 2020; Zhelezoglo et al., 2020). In addition, it has shown the ability to integrate new class members (Arntzen, 2012), remain effective in serial presentations without response requirements (Avellaneda et al., 2016), and persist over time (Regaço et al., 2023). Taken together, these findings underscore the generality of stimulus equivalence learning and its relevance to neuropsychological research.

Drawing on this framework, SEL has over the years gained attention in the field of dementia and rehabilitation (Aggio et al., 2021; Brogård Antonsen & Arntzen, 2023; Cowley et al., 1992; Ducatti & Schmidt, 2016; Sidman, 2013). Given that conditions such as dementia are characterized by behavioral changes that limit the person’s ability to respond to familiar stimuli in functionally appropriate ways (e.g., deficits in recognition and remembering), SEL offers a means to reestablish functional stimulus control. By arranging reinforcement contingencies that promote the emergence of equivalence classes, SEL may help compensate for the loss of discriminatory behavior relations and improve functional outcomes such as daily routines, expressing the correct names for familiar faces, and failure to take medicine at the appropriate time. Although promising, results so far are mixed regarding the extent to which relations could be restored or new relations were formed (Gallagher & Keenan, 2009; Paranhos et al., 2018; Steingrimsdottir & Arntzen, 2014a).

This current scoping review assesses the scope and nature of research on the reapplication of identity matching and stimulus equivalence paradigms for memory rehabilitation (i.e., to improve remembering and relational responding) in individuals suffering from explicit memory dysfunctions. Results aim to provide insights into the paradigms’ potential for addressing everyday deficits and limitations related to stimulus control in this population. Highlighting the benefits, limitations, and common challenges associated with this approach may help to guide future research and identify the preserved aspects of relational learning. Given the expected limited body of available evidence and the methodological variability among studies, we opted to conduct a scoping review (Munn et al., 2018) that addresses the literature gap and identifies

1. The disorders for which there is evidence for the efficacy of SEL and of identity matching in improving memory performance (stimulus control, retention, and remembering), or the formation of equivalence classes;
2. Effective strategies and challenges associated with applying relational learning paradigms in this population; and
3. Evidence-based insights to guide future research and its applicability.

## Methods

The review follows the PRISMA-P guidelines for scoping reviews (Tricco et al., 2018). The protocol has been pre-registered on the Open Science Framework on February 26, 2024, 10.17605/OSF.IO/CZGXF). The population of interest in the present review was defined as patients experiencing memory impairment due to dementia, mild cognitive impairment, brain injury, or related brain disorders with explicit memory dysfunction. Concepts addressed by the review were protocols, techniques, or interventions based on the principle of SEL, using conditional discrimination and MTS tasks to enhance memory performance, such as remembering and recognition. No specifications were made regarding the context.

A search strategy was established in consultation with an academic librarian and a systematic search was conducted in four databases: Web of Science, Medline, Embase, and PsychINFO (a complete search strategy is provided in Appendix 1). Examples of search terms (and truncated versions) included *“Amnesia,” “Dementia,” “Memory Disorders,” “Mild Cognitive Impairment,” “Brain Damage,” “Cerebrovascular Disorders” AND “Cognitive Rehabilitation,” “Memory Training,” “Intervention,” AND “Matching to Sample,” “Conditional Discrimination,” “Stimulus Equivalence,” “Relational Frame Theory.”* Relational frame theory (RFT; Hayes et al., 2001) offers a broader behavior-analytic account of human language and cognition, viewing equivalence as one type of relational responding. Although RFT is not the primary focus of this review, it was included as a search term to capture potentially relevant extensions of SEL within this framework. No date or language restrictions were applied. The final search on all four databases was performed in February 2024. The reference list of all included sources was screened for additional articles of relevance.

### Inclusion and Exclusion Criteria

Studies were evaluated for eligibility based on the following inclusion criteria:

1. Participants of included studies had a diagnosis of any type of dementia, mild cognitive impairment, brain injury, or related brain disorders with explicit memory dysfunction;
2. The study employed a task, protocol, technique, or intervention based on relational learning paradigms such as SEL or identity matching (e.g., conditional discrimination, or MTS);
3. The outcome measure of the study is related to stimulus control, remembering, recognition, every day (functional) skills, or emergent relations; and
4. The study has been peer-reviewed and published.

Exclusion criteria included

1. Reviews;
2. Grey literature and nonpeer-reviewed work; and
3. Unpublished data or conference proceedings.

### Screening and Selection Process

**Fig. 2:**
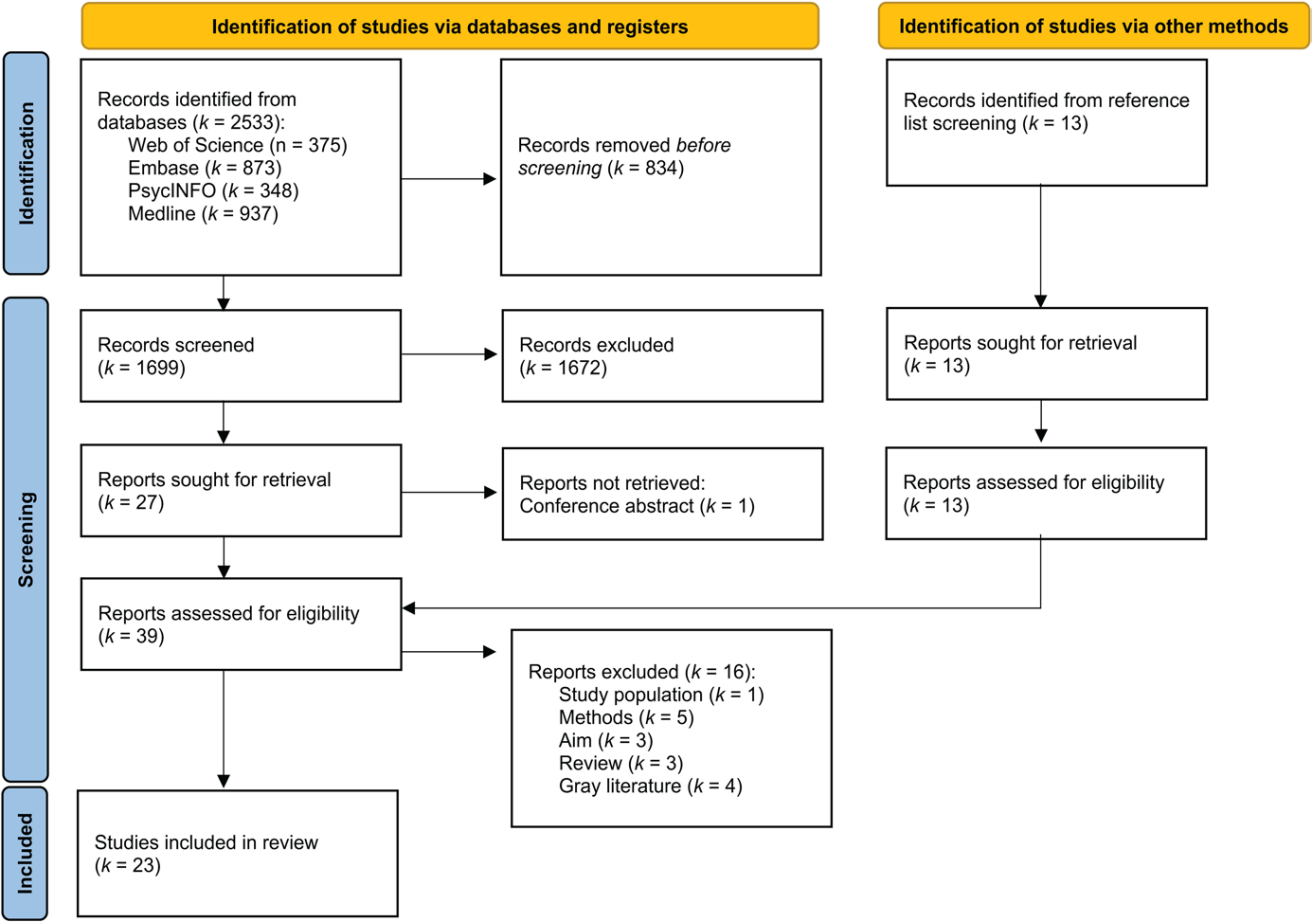
PRISMA Flowchart of the Study Selection Process. *Note.* An initial search across all databases resulted in 2,533 reports. After the deletion of duplicates, abstract, full text, and reference list screening, 39 reports were assessed for eligibility. The exclusion of 16 reports resulted in 23 included studies

All identified sources were exported into Zotero Version 7.0 (Stillman et al., 2024). Using *Rayyan* (http://rayyan.qcri.org; Ouzzani et al., 2016), duplicates were indicated and manually removed. No automation tools were used in the process. Titles and abstracts were screened by the first author for eligibility based on the in- and exclusion criteria. A full-text screening was performed by the first author and validated by the second author. Discrepancies in the full-text screening were resolved by discussion. The selection process, including reasons for exclusion, is presented in Fig. 2. A data extraction template was created, including the variables *author, date, country, aim, sample, task and stimuli, methods (design, procedure, duration, feedback, outcome parameters), *and* main findings.* The variables were chosen in line with the research questions and the aim of the review. A pilot charting was performed with five articles to adjust and validate its feasibility. Thereafter, the data were extracted by the first author and reviewed by the second author, including additional clarification. Disagreements were resolved through discussions. Because of the heterogeneity of the methodology used by the included sources, the evidence was reviewed narratively according to the synthesis without meta-analysis guidelines (Campbell et al., 2020). A summary of the data extraction for each included source is presented in a table format (Tables 1, 2 and 3).

### Quality Assessment

A critical appraisal of the sources was conducted independently by the first author and third author utilizing the Joanna Briggs Institute (JBI) Critical appraisal tools depending on the design of the study:

1. The revised JBI critical appraisal tool for the assessment of risk of bias for quasi-experimental studies (Barker et al., 2024);
2. The JBI critical appraisal checklist for case-reports (Moola et al., 2020);
3. The revised JBI critical appraisal tool for the assessment of risk of bias for randomized controlled trials (Barker et al., 2023); and
4. The JBI critical appraisal checklist for case series (Munn et al., 2020). Discrepancies between the ratings were resolved by discussion.

Three categories were created through discussion to rate the quality of the reports based on the answers to the checklists (“low” if > 3 “No” answers; “moderate” if > 1–3 “No” answers; “high” if 0 “No” answers).

## Results

Across all four databases, 2,533 reports were identified in the final search. Figure 2 shows an overview of the selection procedure. After duplicate deletion of 834 reports, titles and abstracts of 1,712 reports were screened for eligibility. The exclusion of 1,672 reports resulted in 27 reports being retrieved for full-text screening. One report could not be retrieved and was subsequently excluded after being identified as a conference proceeding. In addition, through screening the reference lists of included sources, an additional 13 reports of relevance were identified. Hence, 39 reports entered the full-text screening procedure, during which 16 reports were excluded due to the following reasons:

1. The study population did not match the criteria of the review (*k* = 1);
2. A different method than defined by the criteria was employed (*k* = 5);
3. The aim did not match the criteria (*k* = 3); and
4. The report was a review or grey literature (*k* = 7).

This resulted in 23 reports being included in the present review. The reports were classified into three categories based on the variations of the SEL paradigm utilized:

1. Identity matching;
2. Arbitrary matching; and
3. Differential outcome procedure.

### Identity Matching

**Fig. 3:**
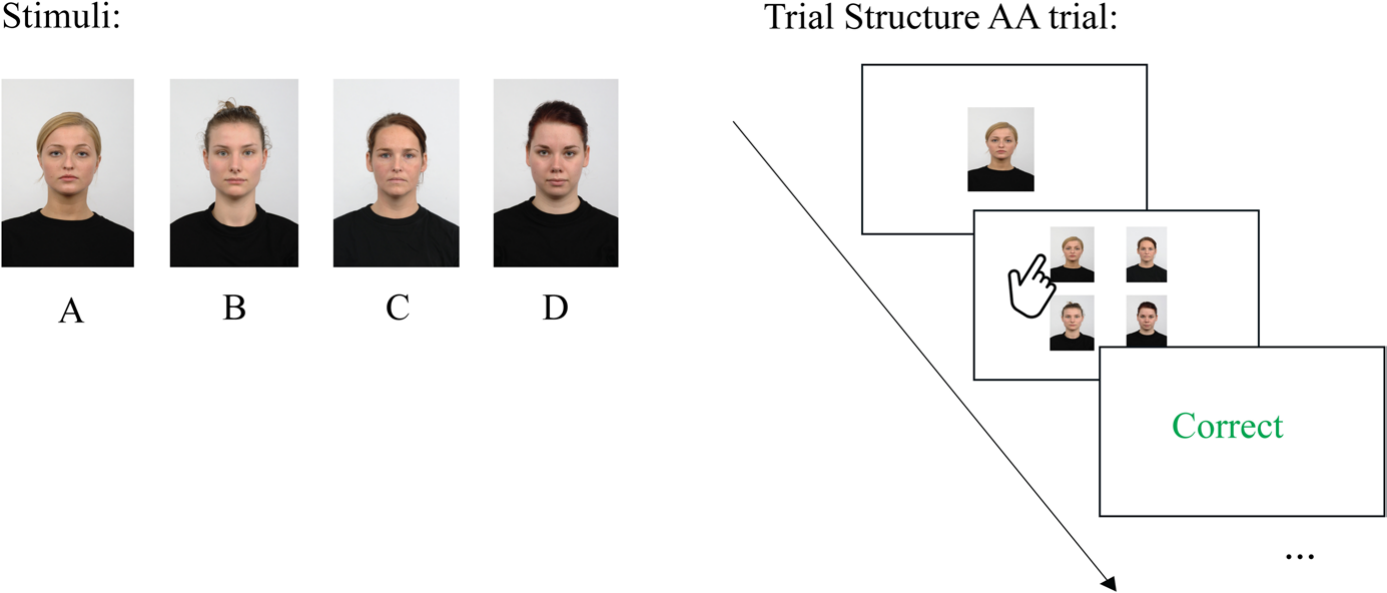
Identity Matching-to-Sample (MTS). *Note.* In identity MTS tasks, participants match a sample stimulus (e.g., stimulus A) to an identical stimulus from a set of comparison stimuli (e.g., A, B, C, and D). The sample is presented first, followed by the sample alongside the comparison stimuli. The task is to select the identical match. Correct choices trigger “correct” feedback, whereas incorrect choices (selecting B, C, or D) result in “false” feedback. Subsequent trials vary the sample (e.g., stimulus B). Task difficulty can be adjusted by modifying the delay between sample and comparisons, the number of comparisons, and their similarity. Performance is measured by the number of correct responses. Face stimuli were sourced from the Radboud Face Database (Langner et al., 2010). This task assesses associative learning, recognition, stimulus control, and basic matching behavior

Identity matching is a basic form of the MTS task, where participants are required to match a given stimulus (the sample) to an identical stimulus within a set of comparison options. The sample serves as the reference item for the participant to match (Fig. 3). For instance, if the sample is a specific face (stimulus A in Fig. 3 left panel), the correct response would be to select the identical face (stimulus A) from the comparison stimuli (stimuli A, B, C, and D; right panel Fig. 3). Feedback is provided after each response to reinforce learning and enhance task accuracy over time. The number of comparison stimuli and their similarity to the sample can be adjusted to modify task difficulty.

As an alternative, within the variant of generalized identity matching, instead of selecting an identical match, participants must demonstrate an understanding of “sameness” between stimuli that share a category or abstract feature. Testing the ability to apply the matching rule beyond specific instances examines flexibility in applying learned associations to novel stimuli. Due to the simplicity of identity matching, there is no need for complex reasoning. Although this variant does not lead to emergent relations, it can assess associative learning, recognition, stimulus control, and basic matching behavior.

Extracted data of each report are presented in Table 1. Of the included reports, five reports employed identity matching with the aims of

1. Examining identity-matching performance as influenced by delay intervals and identifying variables affecting memory (Arntzen et al., 2013; Arntzen & Steingrimsdottir, 2014);
2. Investigating the effect of teaching conditional relations by identity matching on performance (Camara et al., 2017); or
3. Comparing simultaneous and delayed MTS, and assessing the effect of the number of comparisons on performance (Sahakian et al., 1988; Steinunn Steingrimsdottir & Arntzen, 2011).

**Table 1:** Identity Matching: Study Characteristics and Main Findings of Included Studies

IDENTITY MATCHING						
Report	Sample	MMSE	Aim	Task & Stimuli	Methods	Main Findings
Arntzen and Steingrimsdottir, 2014 (Norway)	*N* = 1 Age = 62 M Dementia	23	Examine the effect of different delay values in MTS procedures on identity matching performance	Identity DMTS, computerized *Stimuli:* 3 abstract shapes (color stimuli)	*Design:* Single case; withdrawal ABAB (A: 500ms change in delay; B: 100ms change). Pretest & pretraining Conditional discrimination training & testing *Feedback:* Only during training *Duration:* Multiple sessions *Parameters:* Number of correct responses & RT; Criterion 100% to increase delay; asymptotic value	Small titration steps (100 ms) resulted in smoother response patterns, higher accuracy, and more stable performance, whereas larger steps (500 ms) led to decreased accuracy and greater variability. Matching behavior was not consistently under optimal stimulus control.
Arntzen et al. 2013 (Norway)	*N* = 1 Age = 85 F AD	20	Studying identity matching as a function of delay in DMTS procedures and use those to study variables affecting memory	*Study 1:* Identity MTS (3 comparisons; fixed delay of 12000ms/10000ms; computerized) *Study 2:*identity MTS (3 comparisons; titrating delays; computerized) *Stimuli:* South Park figures	*Design Study 1:* Single case; ABA withdrawal 7 training phases, then testing *Design Study 2:* Single case; observational *Feedback:* 100% feedback during training, then gradual reduction to 0%. *Duration:* Participant determined, multiple sessions *Parameter:* Number of correct responses & RT Criterion 100% to increase delay, end of training 90%	Study 1: Correct responding with 10000ms delay but not 120000ms. Maintenance of correct responding (at 24 hr). Study 2: Highest titration value was 12250ms (no stable responding).
Camara et al. 2017 (Brazil)	*N* = 24 (*n* = 8) Age = 60–92 HC, NCD 1, NCD 2, NCD: AD or nonspecific dementia	HC: 24–30 NCD 1: 9–15 NCD 2: 8–12	Effects of teaching conditional relations by identity MTS on performance (& by exclusion)	(Generalized) identity MTS (3 comparisons, table-top) Exclusion tests *Stimuli:* geometric shapes, colors & characters of the Sesame Street	*Design:* Quasi experimental Pre-tests (group division): accurate performance: Yes (NCD 1), No (NCD 2) MTS training only for NCD 2 Posttests for all groups (Exclusion & Generalized MTS) *Feedback:* Only during training *Duration:* Average of 5 sessions, 30 min each *Parameter:* Number of correct responses, Criterion training: 90%	Positive results of identity matching training (all but one NCD 2 participant reached the criterion). Mixed results for generalized identity matching post-test. No effects of training were observed in the exclusion posttests.
Sahakian et al. 1988 (England)	*N* = 24 (*n*=12) Age (mean) = 72 Group 1: AD Group 2: HC		Comparison of groups on several computerized tests of visuospatial recognition & learning	Identity SMTS / DMTS (with 0,4,8,16s; 4 comparisons, computerized) *Stimuli:* Complex abstract visual pattern	*Design:* Quasi-experimental, between subject design *Feedback:* Visual/auditory for correct & incorrect *Duration:* Single session *Parameter:* Number of correct responses & Criterion 100%	Patient group exhibited a delay-dependent deficit in DMTS but were not impaired at SMTS.
Steinunn Steingrimsdottir and Arntzen, 2011b (Norway)	*N* = 1 Age = 80 M AD	10	1) Comparing SMTS vs. DMTS 2) Assessing the effect of the amount of comparison stimuli	Identity SMTS & DMTS (2–3 comparison; computerized) *Stimuli:* South Park figures	*Design:* Single case, pre- & posttest 4 experimental conditions based on task and comparisons *Feedback:* Only during training & reduction during last phase (75%–0%) *Duration:* Short sessions of ~13 min, across 18 days *Parameter:* Number of correct responses, Criterion training: 90% - 100%	Although not reaching criterion, performance increased through training. Benefit with 0s-delay MTS (2 comparisons). Decreased performance with SMTS (3 comparisons).

MMSE = Mini-Mental State Examination. Scores range from 0 to 30, with higher scores indicating better cognitive function; NCD = Neurocognitive disorder, AD = Alzheimer’s Dementia, HC = Healthy Controls, Min = Minutes, MTS = Matching-to-Sample, DMTS = Delayed MTS, SMTS = Simultaneous MTS, RT = Reaction time

All reports included older patients with cognitive impairments without further diagnostic details (*k* = 4 questionably referred to as “Alzheimer’s disease”; *k* = 1 unspecified dementia (Arntzen & Steingrimsdottir, 2014). Mini-Mental-State-Examination (MMSE; Folstein et al., 1975) scores ranged from 9 to 23, and the participants’ ages spanned 60 to 92 years across all five studies. Three reports were single-case reports (Arntzen et al., 2013; Arntzen & Steingrimsdottir, 2014; Steinunn Steingrimsdottir & Arntzen, 2011), and two reports had a quasi-experimental design (Camara et al., 2017; Sahakian et al., 1988).

The five reports share methodological similarities but also exhibit notable differences. All but one study, which used a paper-and-pencil format (Camara et al., 2017), employed a computerized MTS task. Likewise, all but one study (Camara et al., 2017) employed a delayed MTS task (DMTS). In the DMTS, the sample is first presented on screen alone, and upon its disappearance, with an *n*-s delay, the comparison stimuli are displayed. In addition to DMTS, three studies also included a simultaneous MTS (SMTS) task, in which the sample remains visible alongside the comparison stimuli (Camara et al., 2017; Sahakian et al., 1988; Steinunn Steingrimsdottir & Arntzen, 2011). Apart from DMTS and SMTS procedures, exclusion MTS was also used in one study (Camara et al., 2017). In this procedure, participants are presented with a novel sample stimulus and must select the correct match from comparison stimuli which include a novel comparison (the correct match) and familiar comparisons. The correct match is identified by recognizing the familiar comparisons and distinguishing them from the novel comparison.

Stimuli varied across reports in type and number, ranging from two to four, with most studies using three comparisons. Two studies used cartoon figures (e.g., South Park or Sesame Street characters; Arntzen et al., 2013; Camara et al., 2017; Steinunn Steingrimsdottir & Arntzen, 2011), whereas the rest utilized abstract shapes and patterns (Arntzen & Steingrimsdottir, 2014; Sahakian, et al., 1988). All studies included pre- and posttesting to assess changes in performance and learning, using common outcome measures such as correct responses, reaction time, and a 90% training success criterion. Four reports involved multiple training sessions (Arntzen et al., 2013; Arntzen & Steingrimsdottir, 2014; Camara et al., 2017; Steinunn Steingrimsdottir & Arntzen, 2011), and one report utilized a single session (Sahakian et al., 1988). In all studies, feedback was provided only during training. Two studies gradually reduced feedback to 0% during later training phases to prevent extinction effects during testing (Arntzen et al., 2013; Steinunn Steingrimsdottir & Arntzen, 2011).

The outcomes are mixed. Although there is evidence of successful matching performance, defined by reaching a criterion during testing (Arntzen et al., 2013; Camara et al., 2017; Sahakian et al., 1988), this was not the case in all reports (Arntzen & Steingrimsdottir, 2014; Steinunn Steingrimsdottir & Arntzen, 2011). Moreover, Sahakian et al. (1988) observed a delay-dependent deficit in patients compared to no impairment during SMTS, whereas Steingrimsdottir and Arntzen (2011) found a benefit of 0-s DMTS over SMTS. In summary, although identity matching can result in successful matching performance, no conclusion on its efficacy can be made based on the current state of evidence, because there were profound differences regarding sample characteristics (e.g., a wide range of MMSE scores and ages) and methods (e.g., stimuli and procedures).

### Arbitrary Matching

**Fig. 4:**
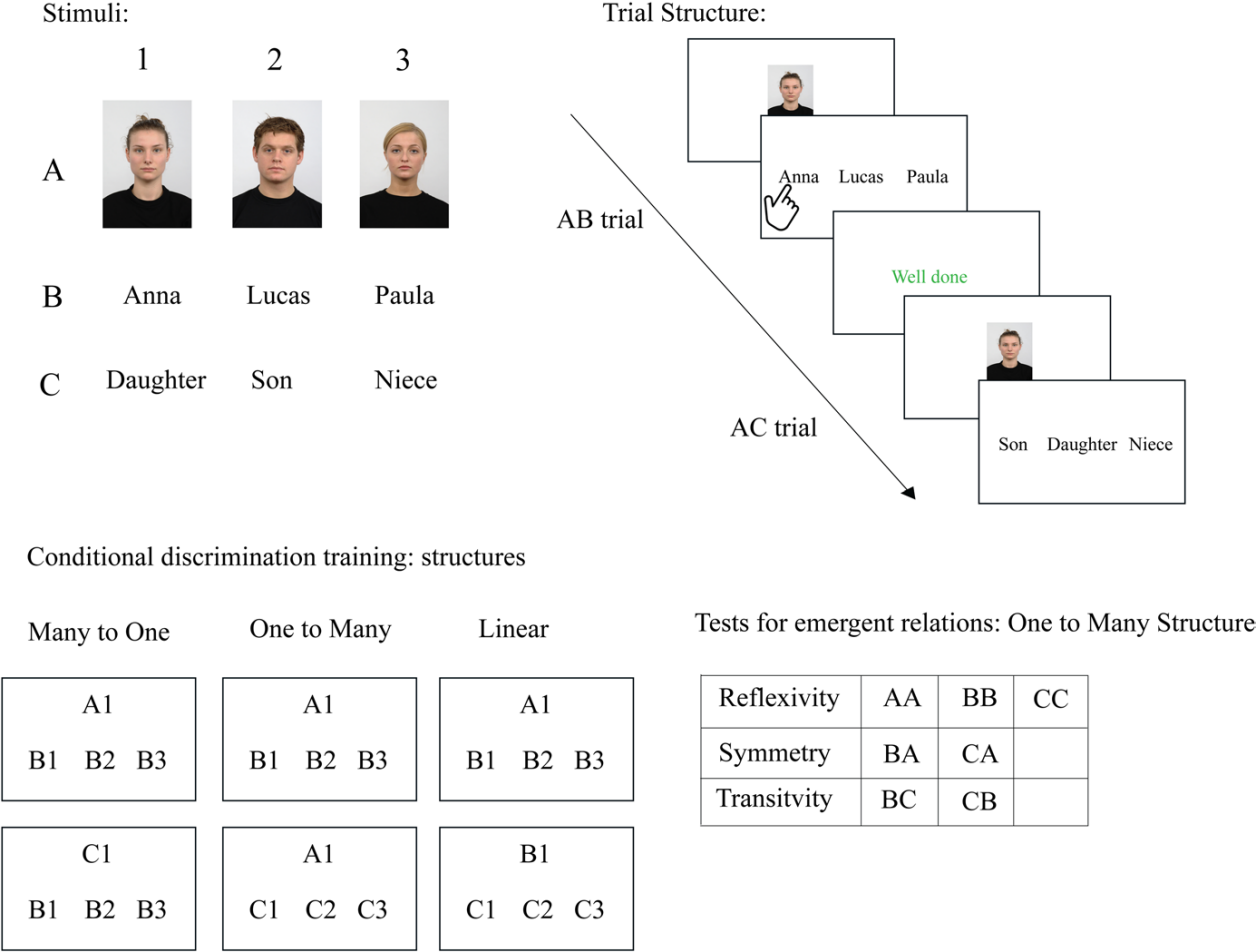
Arbitrary Matching-to-Sample. *Note.* In arbitrary matching-to-sample tasks, stimuli with no inherent similarity are linked through preestablished relations learned during the task, resulting in both conditional and untrained (emergent) relations. The top-left panel illustrates the stimuli (classes and members) assigned to letters and numbers for clarity. Three equivalence classes are shown, such as face A1, name “Anna” (B1), and relation “Daughter” (C1). The top right panel depicts two trial types: in the first, a face (A1) is followed by comparison names (B1, B2, B3), and the participant must select the correct name (B1, “Anna”). In the second, the face (A1) is followed by comparison relations (C1, C2, C3; correct match: C1, “Daughter”), where the correct response is also the matching class member. Feedback is provided for correct or incorrect choices. Training can use different structures, as shown in the bottom-left panel: Many-to-One (MTO; A = B, C = B), One-to-Many (A = B, A = C), and Linear structure (LS; A = B, B = C). After training, participants are tested on both trained and untrained relations (emergent relations) as shown in the bottom right panel. Testing includes reflexivity (A = A, B = B, C = C), symmetry (e.g., B = A, C = A), and transitivity (e.g., B = C, C = B). If participants pass these tests, stimulus equivalence classes (e.g., A1, B1, C1) are considered established

Unlike simpler identity matching, arbitrary matching is used to establish stimulus equivalence classes, where stimuli are functionally grouped despite potential differences in category or modality (Fig. 4). In this paradigm, matching stimuli share no inherent similarity but are linked through preestablished arbitrary associations learned during the task. At its core is the mechanism that if participants learn A matches B and A matches C, they can infer that B also matches C. Conditional discrimination training through arbitrary MTS tasks gives rise to emergent, or untrained, relations (e.g., A = B, A = C implies B = C). Testing of these untrained relations examines reflexivity (A = A, B = B, C = C), symmetry (e.g., B = A, C = A), and transitivity (e.g., B = C, C = B), demonstrating that the paradigm engages not only associative learning but also relational reasoning. The training phase in arbitrary matching can follow different structural approaches, with three commonly used methods: Many-to-One (MTO; A = B, C = B), One-to-Many (OTM; A = B, A = C), and Linear Structure (LS; A = B, B = C).

Arbitrary matching was used to establish stimulus equivalence classes in 14 reports. Table 2 shows the extracted data from each report. Six reports focused on older patients with cognitive impairments but without any further diagnostic details (questionably referred to as “Alzheimer’s disease”; Aggio et al., 2021; Brogård Antonsen & Arntzen, 2023; Brogård-Antonsen & Arntzen, 2019; Ducatti & Schmidt, 2016; Presti et al., 2017; Steingrimsdottir & Arntzen, 2011), three included patients with brain injuries with memory impairment (Cowley et al., 1992; Green, 1991; Guercio et al., 2004), two reported on patients with vascular dementia (Steingrimsdottir et al., 2021; Steingrimsdottir & Arntzen, 2014b), one involved a patient with unspecified dementia (Arntzen et al., 2015), one reported on elderly with low cognitive screening scores (MMSE; Gallagher & Keenan, 2009), and one included stroke patients with memory impairments (Paranhos et al., 2018).

**Table 2:** Arbitrary Matching: Study Characteristics and Main Findings of Included Studies

ARBITRARY MATCHING						
Report	Sample	MMSE	Aim	Task & Stimuli	Methods	Main Findings
Aggio et al. 2021 (Brazil)	*N* = 1 Age = 84 F AD	13	Establishing equivalence classes	Arbitrary SMTS (2–3 comparison; OTM, tabletop) *Stimuli:* Names, Roles, & Persons	*Design:* Single case (pre & post-test) Conditional discrimination training Tests for symmetry & equivalence *Feedback:* Correct both in training & testing; incorrect only during training *Duration:* 3 sessions (each 15–30 min) over 3 weeks *Parameter:* Number of correct responses, Criterion: no more than one error per relation	Established equivalence classes (successful training and testing) with 2 comparison stimuli. 3 comparisons disrupted performance, partly showed transitivity.
Arntzen et al. 2015 (Norway)	*N* =1 Age = 61 F Unspecified dementia	17	Establishing equivalence classes and exploring morphing technique	DMTS (0,3s; 2 comparisons, OTM, computerized) *Stimuli:* name (spoken), name (written), image of face	*Design:* Single case (pre- & posttest) Conditional discrimination training (including morphing procedure of name into image, hierarchical 78% text to 100%image) Tests for symmetry & equivalence *Feedback:* Only during training *Duration:* 62 training days; follow-up 45 days after *Parameters:* Number of correct responses, Criterion: 90%	Increase of correct responding throughout training and participant mastered final step of morphing. No new (untrained) relations emerged.
Brogård-Antonsen and Arntzen, 2019 (Norway)	*N* = 1 Age = 89 F AD	14	Establishing equivalence classes and test for maintenance	SMTS (3 comparison, MTO, computerized) *Stimuli:* Names, Relation, & Portrait	*Design:* Single case (pre- & posttest) Conditional discrimination training Testing for symmetry & equivalence *Feedback:* Only during training & gradual reduction *Duration:* 3 periods with 10–15 min sessions, follow-up at 1 week, 9 months, and 1 year *Parameters:* Number of correct responses, Criterion 90%	Performance increased from Period 2 to 3. Demonstrated stimulus equivalence after period 2 and in follow-up. Did not show symmetry, also not during the follow-up testing.
Brogård-Antonsen and Arntzen, 2023 (Norway)	*N* = 1 Age = 72 M AD	25	Identifying intact relations and reestablishing relation between stimuli	SMTS (3 comparison, MTO, computerized) *Stimuli:* Names, Relation, & Portrait	*Design:* Single case (pre- & posttest) Conditional discrimination training Testing for symmetry & equivalence *Feedback:* Only during training & gradual reduction *Duration:* Single session *Parameters:* Number of correct responses, Criterion 90%	After adjusting the procedure functional skills have been re-learned and equivalence emerged.
Cowley et al. 1992 (USA)	*N* = 3 Age = 45, 30, 57 Brain injuries (Stroke/Diffuse damage/Frontal lobe) with severe memory deficits	/	Test effectiveness of stimulus equivalence methods	SMTS (3 comparison; OTM, table-top) *Stimuli:* Dictated names, faces, written names & nameplates	*Design:* Case series report with pretest/posttest design Naming/sorting application test pre-training Conditional discrimination training Tests for symmetry & equivalence Naming/sorting application test post-training *Feedback:* Only during training *Duration:* 8–13 weeks (3–5 days a week; each session 30–50 min) *Parameter:* Number of correct responses; Criterion: no more than one error per stimulus	All participants demonstrated the formation of three equivalence classes. Application tests (transfer to “real world”) showed limiting but promising data.
Ducatti and Schmidt, 2016 (Brazil)	*Study 1:* *N* = 11 Mean Age = 77.8 / 81.6 HC (*n* = 5) AD (*n* = 6) *Study 2:* *N* = 4 Age = 75-85 AD & mild cognitive impairment	HC = 18–29 AD = 12–17 Study 2: 14–16	*Study 1:* Establishing equivalence classes *Study 2:*Effectiveness of teaching by exclusion	*Study 1:* MTS (4 comparisons; LS, computerized) *Stimuli:* Names,photos, written names, relatedness, and profession *Study 2:* SMTS (2–3 comparisons; OTM) *Stimuli:* Photographs, written names, professions	*Design:* Quasi experimental Naming tests (pre-& posttest) Conditional discrimination training with gradual introduction of comparisons Tests for symmetry & equivalence *Feedback:* Only during training (except end) *Duration:* Multiple sessions *Parameter*: Number correct responses, Criterion 90% *Study 2:* Same procedure but no HC group	*Study 1:* 3/5 HC but no AD patient formed equivalence classes (AD did not learn the conditional relations) *Study 2:* All participants learned conditional relations by teaching by exclusion & delayed cue procedure, but none formed equivalence classes.
Green, 1991 (USA)	*N* = 2 Age = 17 & 20 Brain injuries	NA	Re-teaching food groups through establishing equivalence classes	SMTS (3 comparisons; OTM, table-top) *Stimuli:* Food groups	*Design:* Case series report with pretest-posttest Conditional discrimination training Tests for symmetry & equivalence *Feedback:* Only during training for correct trials (including reduction to 20%) *Duration:* Several weeks each weekday ~40 min *Parameter:* Number correct responses; Criterion: no more than one error on each relation	Equivalence classes have developed (taught 6 relations and showed an additional 12 relations).
Gallagher and Keenan, 2009, (USA)	*Study 1:* *N* = 18 Age = 67–94 Elderly *Study 2:* *N* = 12 Age = 57–92 Elderly *Study 3*: *N* = 15 Age = 69–95 Elderly	*Study 1:*16–30 *Study 2:*21–29 *Study 3:* 27–30	*Study 1:* Establishing stimulus equivalence and relation to MMSE *Study 2 & 3:* Replication	SMTS (2 comparisons; LS; table-top) *Stimuli:* Arbitrary symbols & nonsense syllables	*Design:* Pre- & posttest Conditional discrimination training Tests for symmetry & equivalence *Feedback:* Only during training *Duration:* NA *Parameter:* Number of correct responses, Criterion: 90%	90% who scored 27 or above in the MMSE established equivalence classes. Participants with MMSE < 26 did not. In total 11/18 formed equivalence classes (3/12 in study 2; all 15 in study 3). Correlation between equivalence performance and MMSE scores.
Guercio et al. 2004, (USA)	*N* = 3 Age = 19–27 Brain injuries	GCS 12–13	Teach emotion recognition skill with stimulus equivalence techniques	SMTS (3 comparison; OTM; computerized) *Stimuli:* Dictated word, photos, updated photos (of emotions)	*Design:* Pre- & posttest Conditional discrimination training Tests for symmetry & equivalence *Feedback:* only for correct during training *Duration:* Multiple sessions; each session 15–30 min *Parameter:* Criterion pre- & posttest: 89%	Participants demonstrated increased facial-emotional recognition skills after training.
Paranhos et al. 2018 (Brazil)	*N* = 9 (*n* = 3) Age = 45–60 HC (*n* = 3) Stroke with cognitive impairment Stroke patients without cognitive impairment	HC = 26–30 Stroke with CI = 14–17 Stroke without CI = 27–30 *Study 2:* similar but stroke with CI 20–22	1) Establishing equivalence classes 2) Comparing sensibility of MTS vs. MMSE 3) Verify N400's occurrence	SMTS (3–4 comparison; OTM; computerized) *Stimuli:* Abstract figures	*Design:* Quasi experimental Conditional discrimination training Tests for symmetry & equivalence *Feedback:* Correct & incorrect during part of the training *Duration:* / *Study 2:* EEG before & after MTS training & testing *Parameter:* Number of correct responses; Criterion: 90%	HC and Stroke patients without cognitive impairment formed equivalence classes. None of the patients with cognitive impairment established equivalence classes. High MMSE score (> 27) was consistent with equivalence formation. Study 2 replicated results from study 1.
Presti et al. 2017 (Italy)	*N* = 26 Mean age = 77.8 AD	10–24	Evaluate the efficacy of a multiple-exemplar relational training (RFT) as add-on non-pharmacological therapy	Strengthening Mental Abilities with Relational Training (SMART; Cassidy, Roche B, & Hayes, 2011) program (computerized; OTM), same/opposite; more-than/less-than relations *Stimuli:* nonsense syllables	*Design:* Randomized parallel group study Group 1: Monotherapy (ChEIs) Group 2: Combined therapy (ChEIs + RFT) *Feedback:* Only during training *Duration:* Across 3 months several sessions of 60 min per week *Parameters:* Cognitive/executive functions (cross-sectionally at baseline + end training; Milan Overall Dementia Assessment (MODA), Colored Progressive (CPM) & Attentive Matrices (AM).	Positive effects on MODA & attentive matrices for the combined therapy compared to monotherapy.
Steingrímsdóttir et al. 2021 (Norway)	*N* = 1 Age = 73 M Cerebrovascular dementia	18	Establishing stimulus equivalence classes and assessing maintenance	SMTS (3 comparisons, MTO, STC, & SIM protocols, computerized) *Stimuli:* name, family relation, picture, family members, and pictures of hobbies	*Design:* Single case (pre- & posttest) Conditional discrimination training Tests under different conditions (STC or SIM, 5,000 or 2,000 ms ITI) *Feedback:* Only during training; gradual reduction (75%/50%/25%/0%) only in SIM *Duration:* 3 x week for 5 weeks. Follow-up at 5 weeks and 9 months. *Parameters:* Number of correct responses, Criterion 90%	Correct responding with STC protocol and 5,000 ms ITI. Maintenance at 5 weeks but not at 9-month follow-up. Successful reestablishing of conditional discriminations after repetition of STC protocols with 5,000 ms ITI.
Steingrimsdottir and Arntzen, 2014a (Norway)	*N* = 1 Age = 89 F Vascular dementia	8	1) Studying arbitrary MTS performance 2) Investigate variables influencing establishment of stimulus control	DMTS & SMTS (arbitrary & Identity, 3 comparisons; computerized) *Stimuli:* familiar objects: eating utensils, clothes, hand washing, four colored squares	Design: Single case (pre- & posttest) 12 experimental conditions: arbitrary/identity, simultaneous/delayed, different number of comparisons) *Feedback:* Only during training, gradual decrease (75%, 50% . . .) *Duration:* 10 weeks, 3–4 sessions of maximum 20 min per week *Parameters:* Number of correct responses, Criterion 90%	Arbitrary MTS was not successful in reaching criterion. Correct responding increased with identity MTS with 2 comparisons, specific instructions, and extended training.
Steingrimsdottir and Arntzen, 2011 (Norway)	*N* = 1 Age = 84 F AD	20	Comparing arbitrary vs. Identity MTS and the effect of different delays	DMTS & SMTS (arbitrary & identity, 2–3 comparisons, MTO, 0, 3, 6, 9 s delay, computerized) *Stimuli:* South Park figures	*Design:* Single-case (pre- & posttest) 9 experimental condition consisted of each three experimental phases: (1) arbitrary MTS, (2) identity MTS, (3) identity MTS test *Feedback:* Only during training, gradual decrease *Duration:* Multiple sessions *Parameters:* Number of correct responses, Criterion 90%	No correct responding with arbitrary MTS. Both SMTS and DMTS were successful procedures for identity matching and led to correct responding. Increasing delay led to a decrease of correct responses.

MMSE = Mini-Mental State Examination. Scores range from 0 to 30, with higher scores indicating better cognitive function, GCS = Glasgow Coma Scale. Scores range from 3 to 15 with higher scores indicating less severe brain injury, AD = Alzheimer’s Disease, HC = Healthy Controls, Min = Minutes, MTS = Matching-to-Sample, DMTS = Delayed MTS, SMTS = Simultaneous MTS, OTM = Many to One, MTO = Many to One, LS = Linear structure, STC = Simple to Complex procedure, ITI = Intertrial Interval, RT = Reaction time

Seven reports were single-case studies (Aggio et al., 2021; Arntzen et al., 2015; Brogård Antonsen & Arntzen, 2023; Brogård-Antonsen & Arntzen, 2019; Steingrimsdottir et al., 2021; Steingrimsdottir & Arntzen, 2011, 2014a, b); three involved small samples of 2–3 participants (Cowley et al., 1992; Green, 1991; Guercio et al., 2004), and four included medium-sized samples of 9–26 participants (Ducatti & Schmidt, 2016; Gallagher & Keenan, 2009; Paranhos et al., 2018; Presti et al., 2017). All but one study, which was a randomized parallel-group study (Presti et al., 2017), employed a pretest/posttest design to identify existing relations (pretest) and assess emergent relations and improved performance after training (posttest). Seven out of 14 studies used persons, names, and relationships as stimuli (Aggio et al., 2021; Arntzen et al., 2015; Brogård Antonsen & Arntzen, 2023; Brogård-Antonsen & Arntzen, 2019; Cowley et al., 1992; Ducatti & Schmidt, 2016; Steingrimsdottir et al., 2021), whereas the other seven studies employed various stimuli such as emotions (Guercio et al., 2004), food categories (Green, 1991), objects (Steingrimsdottir & Arntzen, 2014b), cartoon characters (Steingrimsdottir & Arntzen, 2011), abstract figures, and syllables (Gallagher & Keenan, 2009; Paranhos et al., 2018; Presti et al., 2017).

The most common structure for conditional discrimination training was OTM (eight reports; Aggio et al., 2021; Arntzen et al., 2015; Cowley et al., 1992; Ducatti & Schmidt, 2016; Green, 1991; Guercio et al., 2004; Paranhos et al., 2018; Presti et al., 2017), followed by MTO (four reports; Brogård Antonsen & Arntzen, 2023; Brogård-Antonsen & Arntzen, 2019; Steingrimsdottir et al., 2021; Steingrimsdottir & Arntzen, 2011) and LS (two reports; Ducatti & Schmidt, 2016; Gallagher & Keenan, 2009). Although DMTS was prevalent in identity-matching studies, arbitrary-matching studies predominantly used SMTS, with one exception (Arntzen et al., 2015). The number of comparison stimuli ranged from two to four.

The feedback pattern was consistent across studies, with all but one (Aggio et al., 2021) providing feedback only during training. Two studies limited feedback to correct responses only (Green, 1991; Guercio et al., 2004). Furthermore, six reports gradually reduced feedback during the final training phase from 100% to 0% (Brogård Antonsen & Arntzen, 2023; Brogård-Antonsen & Arntzen, 2019; Green, 1991; Steingrimsdottir et al., 2021; Steingrimsdottir & Arntzen, 2011, 2014a). Such a gradual decrease in feedback avoids potential extinction effects during the testing phase. Extinction effects can occur after a sudden transition from 100% to 0% feedback. Participants become uncertain and learned relations decrease or disappear because feedback is no longer provided. Therefore, gradually decreasing the feedback before the testing phase ensures stimulus control, meaning that participants are responding based on learned associations rather than reliance on feedback (Aggio et al., 2021; Arntzen et al., 2015; Ducatti & Schmidt, 2016; Sidman & Tailby, 1982).

The reports align on the main outcome parameters, specifically the number of correct responses and an approximate 90% training success criterion. One study was limited to a single session (Brogård Antonsen & Arntzen, 2023), and two did not specify the number of sessions (Gallagher & Keenan, 2009; Paranhos et al., 2018). The remaining studies employed multiple sessions, spanning durations from weeks to months. In addition, three studies included follow-up sessions weeks or months after initial training and testing to assess maintenance (Arntzen et al., 2015; Brogård-Antonsen & Arntzen, 2019; Steingrimsdottir et al., 2021).

Summarizing the main findings, 8 out of 14 studies reported that arbitrary matching successfully or partially established equivalence relations (Aggio et al., 2021; Brogård Antonsen & Arntzen, 2023; Brogård-Antonsen & Arntzen, 2019; Cowley et al., 1992; Green, 1991; Guercio et al., 2004; Presti et al., 2017; Steingrimsdottir et al., 2021). The remaining six studies showed successful learning of conditional relations, but no emergence of untrained relations (Arntzen et al., 2015; Ducatti & Schmidt, 2016; Gallagher & Keenan, 2009; Paranhos et al., 2018; Steingrimsdottir & Arntzen, 2011, 2014a). In particular, procedural adjustments such as a reduction of comparisons, extended training, specific instructions, shortened delay (Steingrimsdottir & Arntzen, 2011, 2014a), teaching by exclusion (Ducatti & Schmidt, 2016), and a morphing technique (Arntzen et al., 2015) improved learning of conditional relations. Last, two studies pointed towards a relation between high MMSE scores and the ability to form equivalence classes (Gallagher & Keenan, 2009; Paranhos et al., 2018).

### Differential Outcome Procedure

**Fig. 5:**
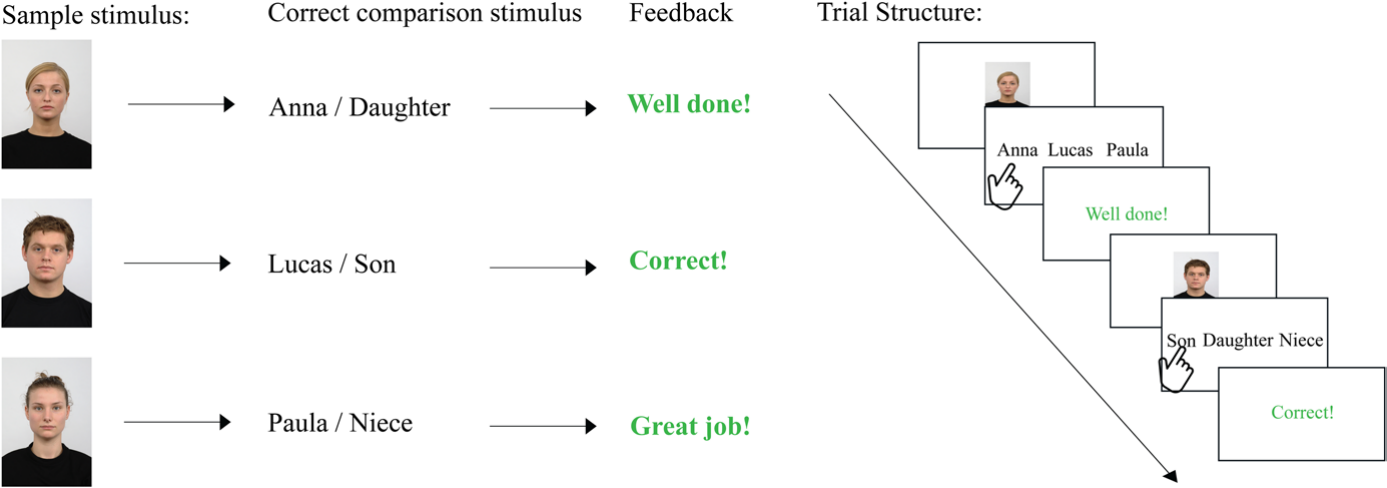
Differential Outcome Procedure. *Note.* The differential outcome procedure (DOP) is a reinforcement method that can be used in combination with identity matching (Fig. 3) and arbitrary matching-to-sample (Fig. 4). Instead of providing generic reinforcement for correct responses (e.g., well done), each stimulus class is assigned a unique reinforcer which becomes part of the stimulus class and, with time becomes functionally equivalent to other stimuli in that class. The left panel illustrates different sample stimuli, their comparisons, and the associated reinforcer. The right panel depicts a trial structure as in an arbitrary matching task but reinforcement according to DOP. Upon seeing a sample stimulus, the participant selects the matching stimulus from the comparison options. If correct, they receive feedback specific to that stimulus class. Reinforcement can include verbal praise as well as tangible rewards such as coins, points, or items

The final category includes studies that employed a specific reinforcement procedure known as the differential outcome procedure (DOP) alongside identity or arbitrary matching (see Fig. 5). In the DOP (Peterson & Trapold, 1980), each stimulus class is assigned a unique reinforcer, which becomes part of the stimulus class and, over time, becomes functionally equivalent to other stimuli in that class. According to Sidman’s theory of equivalence relations (Sidman, 2000), in standard MTS approaches, a common reinforcer acts as a class merger, initially forming one larger class that must thereafter be separated into specific classes (class partition). In contrast, by employing specific reinforcers, class merging is avoided and thereby also the need for class partition. It is interesting that research supports this theory, showing that the DOP facilitates the formation of equivalence classes, improves retention accuracy, and enhances the maintenance of correct responding (McCormack et al., 2019).

This advantage of the DOP over non-DOP approaches aligns with the findings of reports included in the present review (Carmona et al., 2019; Hochhalter et al., 2001; Molina et al., 2020; Plaza et al., 2012). Although not testing for emergent relations, all four reports, which included DOP, reported improved outcomes of retention and recognition under the DOP condition (Table 3). In particular, they examined the effect of DOP on learning and retention of medical information (Molina et al., 2020), facial recognition (Hochhalter et al., 2001; Plaza et al., 2012), and visual recognition and retention (Carmona et al., 2019). Although three reports included patients with Alzheimer’s dementia, one reported from patients with alcohol-related dementia (Hochhalter et al., 2001). All studies employed a quasi-experimental design with medium sample sizes (*N* = 8–20) that included a control group of healthy subjects and pre- and posttests. Feedback was manipulated within subjects by administering either a DOP or non-DOP procedure. Performance was evaluated in all studies based on the percentage of correct responses and reaction time. All but one (Molina et al., 2020) used identity DMTS with two to three comparison stimuli. Variability was seen in stimuli used, which included pill/time of day associations (Molina et al., 2020), faces/person associations (Hochhalter et al., 2001; Plaza et al., 2012), or objects and landscapes (Carmona et al., 2019).

**Table 3:** Differential Outcome Procedure: Study Characteristics and Main Findings of Included Studies

DIFFERENTIAL OUTCOME PROCEDURE						
Report	Sample	MMSE	Aim	Task & Stimuli	Methods	Main Findings
Carmona et al. 2019 (Spain)	*N* = 20 (n = 10) Age = 81 AD, HC	< 20	Using DOP to improve visual recognition in AD and testing long-term retention	Identity DMTS (2- 15s, 2 comparisons, computerized) *Stimuli:* photos of daily objects & natural landscapes	*Design:* Quasi experimental / mixed\within-subject: DOP / Non-DOP Recognition memory post-test *Feedback:* Congratulation phrases for correct (+ landscape picture), blank for incorrect *Duration:* 1 session per condition, 1 week apart; follow-up at 1 hr and 1 week *Parameters:* Percentage of correct responses, median RT, % of hits/ false alarm, discriminability score (d′ = Z hits rate-Z false alarms rate)	Better matching performance and a higher long-term retention of the information when trained under the DOP, relative to the non-DOP. Long-lasting memory retention benefit with the DOP training in AD. No testing for emergent relations.
Hochhalter et al. 2001 (USA)	*N* = 8 (*n* = 4) Age = 64–86 Alcohol-Induced Persisting Dementia, HC	14–28	Testing usefulness of intervention for mediating short-term memory deficits	Identity DMTS (5, 5, 10, 25 s; 2 comparisons, computerized) *Stimuli:* photos of male and female faces	*Design:* Quasi experimental Within-subject: DOP / Non-DOP *Feedback*: both learning & testing (nickels & points) *Duration:* Multiple sessions *Parameter:* Percentage of correct matches Criterion 9/10	Alcohol related dementia group performed more accurately under DOP than non-DOP (no difference for HC). Face-recognition deficits less apparent under DOP condition. No testing for emergent relations.
Molina et al. 2020 (Spain/Chile)	*N* = 19 (*n* = 8) Mean age = 74 AD, HC	19.4 (Mean)	Effectiveness of DOP in learning and retention of medical information in AD	Arbitrary DMTS (2 comparisons, computerized) *Stimuli:* different medication + morning & night picture	*Design:* Quasi experimental Within-subject: DOP / Non-DOP *Feedback:* Picture of reinforcer after correct trials, blank after incorrect, only during training *Duration:* 1–2 days between trainings, 2 phases (separated by 1 week) with each 3 sessions, each 30 min *Parameter:* Percentage of correct responses & latency	DOP improved learning and long-term retention of two pill/time of day associations. No testing for emergent relations.
Plaza et al. 2012 (Spain)	*N* = 16 (*n* = 8) Mean age = 75/76 AD, HC	Mean AD = 17 Mean HC = 29	Improving facial recognition with DOP	Identity DMTS (5, 25 s; 3 comparisons, computerized) *Stimuli:* photos of men in suits	*Design:* Quasi experimental Within-subject: DOP / Non-DOP *Feedback:* photos of prizes (scarf/mug) for correct responses *Duration:* one session per condition, 1 week apart *Parameters:* Percentage of correct responses, median correct RT	DOP improved delayed face recognition in AD patients (both accuracy and latency and even at the longer delay). Under non-DOP AD patients showed memory decline as usual. No testing for emergent relations.

MMSE = Mini-Mental State Examination. Scores range from 0 to 30, with higher scores indicating better cognitive function, AD = Alzheimer’s Dementia, HC = Healthy Controls, DOP = Differential Outcome Procedure, MTS = Matching-to-Sample, DMTS = Delayed MTS, SMTS = Simultaneous MTS, RT = Reaction time

Taken together, the four reports demonstrate that DOP is an effective, easy-to-implement technique for teaching relations such as face-name or medication-daytime in patients with Alzheimer’s dementia and alcohol-related dementia.

### Quality Assessment

Most reports were rated as low quality (*k* = 15), with seven classified as moderate, and only one as high quality. The result of the quality assessment of each report together with the employed paradigms and outcomes is presented in Table 4. Assessment criteria included participant selection and reporting, experimental design and control, statistical analyses, and conclusion reporting.

**Table 4:** Paradigm, Outcome Overview, and Quality Assessment Outcome per Report

Report	Paradigm	Outcome	Quality
Carmona et al. 2019	Identity DMTS + DOP	+	Moderate
Hochhalter et al. 2001	Identity DMTS + DOP	+	Moderate
Molina et al. 2020	Arbitrary DMTS+ DOP	+	High
Plaza et al. 2012	Identity + DOP	+	Moderate
Arntzen and Steingrimsdottir, 2014	Identity DMTS	+/-	Low
Arntzen et al. 2013	Identity DMTS	+	Low
Sahakian et al. 1988	Identity D/SMTS	+/-	Moderate
Camara et al. 2017	Identity MTS	+	Low
Steingrimsdottir and Arntzen 2011	Identity D/SMTS	+/-	Low
Aggio et al. 2021	Arbitrary SMTS	+/-	Low
Arntzen et al. 2015	Arbitrary DMTS	+/-	Low
Brogård-Antonsen and Arntzen, 2019	Arbitrary SMTS	+	Low
Brogård-Antonsen and Arntzen, 2023	Arbitrary SMTS	+	Low
Cowley et al. 1992	Arbitrary SMTS	+	Low
Ducatti and Schmidt, 2016	Arbitrary SMTS	+/-	Moderate
Guercio et al. 2004	Arbitrary SMTS	+	Low
Green, 1991	Arbitrary SMTS	+	Low
Gallagher and Keenan 2009	Arbitrary SMTS	+/-	Moderate
Paranhos et al. 2018	Arbitrary SMTS	+/-	Moderate
Presti et al. 2017	Arbitrary MTS	+	Low
Steingrímsdóttir et al. 2021	Arbitrary SMTS	+	Low
Steingrimsdóttir and Arntzen, 2014a	Identity & Arbitrary D/SMTS	+/-	Low
Steingrimsdóttir and Arntzen, 2011	Identity & Arbitrary D/SMTS	+/-	Low

Reports are sorted by paradigm. DMTS = Delayed Matching to Sample; SMTS = Simultaneous Matching to Sample; DOP = Differential Outcome Procedure. The column “outcome” refers to the establishment of stimulus equivalence classes (arbitrary MTS), or conditional relations (identity MTS). For “+/-,” conditional relations and partially equivalences classes have been established. The quality assessment was performed by the first and third authors and based on the Joanna Briggs Institute (JBI) Critical appraisal tools depending on the design of the study. The results of the checklist were either “low,” “moderate,” or “high” based on the number of “No” answers (e.g., > 3 = low; 1–3 = moderate; 0 = high)

The assessment emphasizes insufficient patient reporting and missing diagnostic information, which limits the comparability and generalizability of findings. Without clear diagnostic criteria and clinical details, it remains uncertain whether participants had the specified condition and what their cognitive status was at the time of the experiment. Diagnoses should follow standardized guidelines and be reported with sufficient detail, including supporting diagnostic tests and assessments to objectively describe patients' clinical conditions. Clear patient reporting is particularly crucial in single-case studies to allow meaningful comparisons between cases. However, all 10 case reports lacked adequate patient information.

Beyond patient reporting, several study design weaknesses were evident. Poor experimental control and limited internal validity were largely due to procedural adjustments and inconsistent conditions throughout the experiments (Brogard-Antonsen & Arntzen, 2023; Steingrimsdottir & Arntzen, 2011, 2014a). In addition, age ranges were broad and control groups were not matched for age and education, introducing potential confounds (Ducatti & Schmidt, 2016). Although such procedural adjustments can compromise experimental control, they are sometimes necessary to accommodate clinical populations and can be beneficial in an exploratory context.

The assessment also highlighted inappropriate statistical analyses or a lack thereof, and a lack of power analyses, except for Molina et al. (2020). Parametric tests have been conducted on very small samples with no testing for assumptions. Many studies relied on numerical comparisons rather than inferential statistics. For example, reaction times were compared between conditions without statistical validation (Arntzen & Steingrimsdottir, 2014), and high correlations were reported without a supporting correlation analysis (Gallagher & Keenan, 2009). Although the lack of inferential statistics limits the interpretability, it may reflect the exploratory nature of early work in this area, especially in studies with small sample sizes.

In summary, insufficient patient reporting, limited experimental control, inadequate comparison groups, and a lack of application tests and follow-ups contribute to an overall low methodological quality of the included reports. As a result, the findings remain exploratory, and no firm conclusions can be drawn about the paradigm’s efficacy and applicability in this population. However, contextual constraints within this clinical group likely account for some design and sample size limitations. In such cases, detailed reporting and good experimental control become essential. Replications of current findings using well-controlled single-case experimental designs and larger samples with appropriate statistical analyses would be valuable in advancing this research area.

## Discussion

This review aimed to evaluate the scope and nature of research on the application of relational learning paradigms such as SEL and identity matching to improve remembering and relational responding (i.e., memory rehabilitation) in individuals suffering from deficits in remembering and stimulus control. We identified

1. The disorders for which there is evidence for the efficacy of SEL and of identity matching in improving memory performance (stimulus control, retention, and remembering), or the formation of equivalence classes;
2. Effective strategies and challenges associated with applying relational learning paradigms in this population; and
3. Insights to guide future research and its applicability. Studies were categorized into the relational paradigms of identity matching, arbitrary matching, and the differential outcome procedure.

Findings across paradigms were mixed regarding the success of training procedures and the establishment of stimulus equivalence classes. In each paradigm, Alzheimer’s dementia was the most frequently studied condition. However, significant variability in sample characteristics and methodology, along with notable shortcomings in patient and diagnostic reporting, limits the reliability of conclusions about the efficacy and application in this population. Despite these limitations, the exploratory evidence thus far suggests that the efficacy of relational paradigms, especially SEL, depends on the severity of the deficits. Individuals with more severe deficits require greater procedural adjustments and simpler task designs (Aggio et al., 2021; Brogård Antonsen & Arntzen, 2023; Brogård-Antonsen & Arntzen, 2019; Sidman, 2013; Steingrimsdottir & Arntzen, 2014a). The following sections discuss effective strategies, challenges, and directions for future research on its clinical applicability.

Regarding effective strategies, several modifiable variables within the paradigms can enhance learning in this population, including the number of comparison stimuli and their timing (DMTS vs. SMTS). Studies suggest using two to four comparisons is effective, with fewer comparisons making the task easier. Identity MTS studies predominantly used DMTS, whereas arbitrary MTS studies favored SMTS. SMTS was particularly effective in establishing equivalence classes, especially when combined with one-to-many (OTM) or many-to-one (MTO) structures. Although OTM was the most frequently used structure, its effectiveness was comparable to MTO, whereas linear structures proved less effective (Steingrimsdottir & Arntzen, 2011; Ducatti & Schmidt, 2016; Gallagher & Keenan, 2009). However, with only one study employing DMTS, its role in establishing equivalence classes remains unclear within the context of dementia and related disorders with impairments in learning and remembering. In identity matching, however, DMTS may serve as a measure of retention, delay thresholds for correct responding, because it allows for comparisons across different delay lengths (Arntzen et al., 2013; Arntzen & Steingrimsdottir, 2014; Sahakian et al., 1988; Steinunn Steingrimsdottir & Arntzen, 2011).

Optimizing learning conditions through error prevention and reinforcement strategies also seemed to enhance the effectiveness of arbitrary MTS in establishing equivalence classes in this specific patient population. Gradually introducing stimuli, teaching by exclusion, implementing morphing techniques, and progressively increasing task complexity improved outcomes by minimizing errors (Arntzen et al., 2015; Camara et al., 2017; Ducatti & Schmidt, 2016; Steingrimsdottir et al., 2021). Error prevention is a key principle in memory rehabilitation and is also used in vanishing cues and spaced retrieval techniques (de Werd et al., 2013; Kessels & de Haan, 2003; Oudman et al., 2015). Another effective strategy in the context of explicit memory dysfunction involves gradually reducing feedback at the end of training to avoid extinction effects that can result from an abrupt shift from 100% to 0% feedback (Aggio et al., 2021; Arntzen et al., 2015; Ducatti & Schmidt, 2016; Sidman & Tailby, 1982). Eliminating feedback during testing ensures that responses are independent of reinforcement, enhancing stimulus control and generalization, which strengthens the durability of learned relations. Overall, despite high variability across studies, there is some consensus on effective procedures for patients with explicit memory dysfunction, particularly regarding comparison stimuli, task timing, and reinforcement strategies.

Furthermore, the differential outcome procedure (DOP) proved to be an effective complementary strategy for both identity and arbitrary matching in the reviewed studies. Four moderate to high-quality studies demonstrated its benefits, suggesting that DOP can enhance learning and retention accuracy. According to Sidman’s theory of equivalence relations (Sidman, 2000), unique reinforcers become part of the stimulus class, thereby eliminating the need for class partitioning, unlike a common reinforcer, which functions as a class merger. Its efficacy in dementia may suggest that deficits are not in class formation or merging, but in the partitioning of classes when common reinforcers are used. By embedding unique reinforcers within the class structure, the DOP may thus circumvent this impairment and support the reestablishment of functional relations in patients with dementia. Therefore, future research should examine the effect of DOP on emergent relations in light of Sidman’s theory, because this was not part of the studies reviewed here.

Developing effective clinical applications requires careful consideration of the challenges and limitations associated with this population. Patients with more severe deficits, such as in later stages of dementia, seem to benefit from simpler procedures, shorter sessions, and more specific instructions (Camara et al., 2017; Steinunn Steingrimsdottir & Arntzen, 2011). Short attention spans and fatigue further restrict both session duration and frequency (Aggio et al., 2021; Arntzen et al., 2015; Brogård-Antonsen & Arntzen, 2019). When assessing long-term maintenance, potential disease progression must also be accounted for. Although it is crucial to test the persistence of training effects, follow-up assessments should be conducted within a timeframe that minimizes confounding effects of disease progression (Arntzen et al., 2013; Arntzen & Steingrimsdottir, 2014).

Disease progression and severity of the impairment might restrict the choice of applicable paradigms. In line with this, two of the reviewed studies switched from arbitrary matching to simpler identity matching due to patients’ impairments (Steingrimsdottir & Arntzen, 2011, 2014a). However, Steingrimsdottir and Arntzen (2011) also observed challenges in transitioning from identity matching to arbitrary matching, because patients often continued searching for similarities among stimuli in the arbitrary task. Future research could systematically investigate the efficacy of different paradigms across varying severity levels of impairment.

A correlation between the success of the procedure and impairment severity was observed in three reports (Gallagher & Keenan, 2009; Paranhos et al., 2018; Steingrimsdottir & Arntzen, 2011). All three reported that patients with MMSE scores below 27 tended to struggle with forming equivalence classes, instead learning only the directly taught conditional relations. If replicated through well-controlled experiments and if appropriately statistically robust analyses, this finding could suggest a potential assessment tool for relational learning. Such computerized training and testing would be easy to administer, highly accurate, easily scored, and repeatable over time.

Due to limitations in experimental control, patient reporting, and at times statistical analyses, current evidence for the efficacy of relational learning paradigms in the context of dementia and impairments in remembering remains largely exploratory. However, the findings offer valuable insights that can inform future research in this direction. Future studies should focus on identifying the necessary and sufficient conditions for teaching stimulus equivalence relations in this population (Ducatti & Schmidt, 2016). This requires well-controlled experimental designs and detailed reporting to validate the preliminary findings and assess the potential as a clinical intervention. Depending on the design, methodological improvements should include pre- and posttests, matched comparison groups, application tests to evaluate transfer and generalization, and follow-up assessments to measure durability and maintenance.

To enhance real-world applicability, research should examine how stimulus function, significance, motivational factors, and the environmental context influence relational learning and the formation of equivalence classes. Personal or functionally relevant stimuli, such as familiar faces or medications, may increase salience and facilitate stimulus control. Other relevant factors include training duration, timing, and frequency, trainer identity, and modality (e.g., virtual reality, serious gaming), reinforcement type and schedules, self-efficacy, and individual characteristics. Systematic manipulation of these variables, for example, through single-case experimental designs with repeated measurements, may help identify conditions that optimize learning and maintenance. Beyond the formation of relations, research should also explore how these paradigms could support the establishment and maintenance of daily routines, a common challenge in this population. Such research is important to function as a bridge between fundamental relational research and its application in clinical settings.

In conclusion, exploratory evidence suggests that relational learning paradigms, including SEL, may be a promising approach for teaching everyday relations, such as faces, names, and relationships, through arbitrary MTS tasks. By establishing conditional relations, untrained relations can emerge despite the absence of reinforcement, forming stimulus equivalence classes. Identity matching and SEL could serve as rehabilitation tools, counteracting the behavioral changes commonly observed in the context of dementia, and restoring functional skills in everyday tasks. Studies indicate particularly positive outcomes when relational learning is combined with differential reinforcement. However, to fully evaluate its potential, future research must focus on well-controlled studies that include transfer and application tests to assess its efficacy and long-term benefits for this population.

## Data Availability

The current study is a review of previously published data. Additional information associated with this article is available from the corresponding author upon request.

## Appendix 1

Example of the Complete Search Strategy in PsychINFO.

Example: PsychINFO – search 4: 348 records.

**Table:**

1	exp Amnesia/ or exp Dementia/ or exp "Neurocognitive Disorders"/ or "Memory Disorders"/ or "Mild Cognitive Impairment"/ or "Korsakoff Syndrome"/ or "Huntington's Disease"/ or "Kluver Bucy Syndrome"/ or "Older Adulthood"/ or Aging/ or "Brain Damage"/ or exp "Brain Injuries"/ or exp Encephalopathies/ or exp "Cerebrovascular Disorders"/ or exp "Cerebral Ischemia"/ or (amnes* or dement* or alzheimer* or pseudodement* or "frontotemporal lobar degeneration" or "neurocognitive disorder*" or NCD* or ((memory or cognitive* or neuropsychological*) adj1 (disorder* or deficit* or declin* or problem* or impair* or loss)) or "MCI" or Korsakoff* or Huntington* or "Kluver-Bucy" or "Lewy Body" or elder* or "old* adult*" or "aging" or "ageing" or encephalopath* or ((brain or cerebral or head) adj1 (damage* or injur* or trauma*)) or stroke* or "cerebrovascular accident*" or "cva" or "cvas" or "vascular accident*").ti,ab,id.
2	exp "Cognitive Rehabilitation"/ or exp "Neurorehabilitation"/ or Rehabilitation/ or "Memory Training"/ or (rehabilitat* or neurorehabilitat* or training or intervention* or teach* or recover*).ti,ab,id.
3	"Matching to Sample"/ or "Discrimination Learning"/ or "Concept Formation"/ or "Cognitive Generalization"/ or "Stimulus Generalization"/ or "Stimulus Discrimination"/ or "Relational Frame Theory"/ or ("conditional discrimination*" or "discriminati* learning" or "matching-to-sample" or "match-to-sample" or "stimulus equivalence" or "equivalent stimuli" or "identity matching" or "emergent relation*" or "equivalence class*" or "class formation" or "class acquisition" or "concept formation" or "concept acquisition" or "stimulus relation*" or "conditional relation*" or "trained relation*" or "untrained relation*" or "intact relation*" or "arbitrary relation*" or "derived relation*" or "MTO" or "OTM" or "simple-to-complex" or "relational frame theory").ti,ab,id.
4	1 and 2 and 3
